# Comparison of *spa* Types, SCC*mec* Types and Antimicrobial Resistance Profiles of MRSA Isolated from Turkeys at Farm, Slaughter and from Retail Meat Indicates Transmission along the Production Chain

**DOI:** 10.1371/journal.pone.0096308

**Published:** 2014-05-01

**Authors:** Birgit Vossenkuhl, Jörgen Brandt, Alexandra Fetsch, Annemarie Käsbohrer, Britta Kraushaar, Katja Alt, Bernd-Alois Tenhagen

**Affiliations:** Federal Institute for Risk Assessment, Berlin, Germany; Rockefeller University, United States of America

## Abstract

The prevalence of MRSA in the turkey meat production chain in Germany was estimated within the national monitoring for zoonotic agents in 2010. In total 22/112 (19.6%) dust samples from turkey farms, 235/359 (65.5%) swabs from turkey carcasses after slaughter and 147/460 (32.0%) turkey meat samples at retail were tested positive for MRSA. The specific distributions of *spa* types, SCC*mec* types and antimicrobial resistance profiles of MRSA isolated from these three different origins were compared using chi square statistics and the proportional similarity index (Czekanowski index). No significant differences between *spa* types, SCC*mec* types and antimicrobial resistance profiles of MRSA from different steps of the German turkey meat production chain were observed using Chi-Square test statistics. The Czekanowski index which can obtain values between 0 (no similarity) and 1 (perfect agreement) was consistently high (0.79–0.86) for the distribution of *spa* types and SCC*mec* types between the different processing stages indicating high degrees of similarity. The comparison of antimicrobial resistance profiles between the different process steps revealed the lowest Czekanowski index values (0.42–0.56). However, the Czekanowski index values were substantially higher than the index when isolates from the turkey meat production chain were compared to isolates from wild boar meat (0.13–0.19), an example of a separated population of MRSA used as control group. This result indicates that the proposed statistical method is valid to detect existing differences in the distribution of the tested characteristics of MRSA. The degree of similarity in the distribution of *spa* types, SCC*mec* types and antimicrobial resistance profiles between MRSA isolates from different process stages of turkey meat production may reflect MRSA transmission along the chain.

## Introduction

*Staphylococcus (S.) aureus* is a common cause of food poisoning due to the production of various enterotoxins. *S. aureus* is a frequent colonizer of the skin and mucous membranes and therefore, personnel and food-producing animals are the main sources of *S. aureus* in food [1]. The control of *S. aureus* is routinely considered in the food producing industry if standard food safety management systems are operated. In recent years, methicillin-resistant *Staphylococcus aureus* (MRSA), previously known as a multidrug resistant pathogen causing severe healthcare associated and community acquired infections, [2] has been observed worldwide in livestock husbandry as well as in food of different animal origins raising concerns about a possible farm to fork transmission.

First reported from pigs in the Netherlands [3] and France [4] a distinct MRSA lineage, Clonal Complex (CC) 398, has emerged in food producing animals in Europe especially in herds of pigs [5]–[8], veal calves [9] broiler flocks [10], [11] and turkeys [12]. Therefore, the term “livestock-associated MRSA” (LA-MRSA) was introduced considering livestock to form a new and separate reservoir for MRSA [13]. In Asian countries, however, sequence type ST9, a separate genetic linage, is predominating among MRSA isolates from livestock animals [14], [15]. Different DNA sequencing methods are used for typing MRSA strains. In order to define MRSA clones, Multilocus sequence typing (MLST), a method of classifying MRSA strains by the allelic profile of seven housekeeping genes, is used in conjunction with PCR analysis of the staphylococcal chromosomal cassette *mec* (SCC*mec*), a mobile genetic element that contains the *mec A* gene encoding for resistance to methicillin [16]. 11 different SCC*mec* types have been described, so far. The class of *mec* gene complex and the type of ccr gene complex carrying a set of recombinase genes responsible for integration and excision of the cassette characterize the different types of SCCmec elements [17]. Whereas SCCmec I-X harbor *mec*A SCCmec XI carries a divergent *mecA* homologue (*mecA*_LGA251_) [18]. *Spa* typing differentiates MRSA strains by the number of tandem repeats and the sequence variation in region X of the protein A gene (spa) and can be used for reliable and discriminatory typing of MRSA [19]. As particular MLST have shown to be associated with specific repeats and repeat successions it is, with few exceptions, possible to infer an MLST type from the spa type. (http://www.spaserver.ridom.de). The frequent use of antimicrobials in animal production is suspected to facilitate the emergence and spread of MRSA due to antimicrobial selection pressure [20]–[22]. High stocking density in intensive food animal production holdings and intensive animal trading promote the rapid spread of MRSA between livestock populations [23], [24]. LA-MRSA strains have also been detected in raw meat at retail including beef, veal, pork and poultry [25]–[33] indicating potential transmission along the chain due to cross contamination during slaughter and processing. However, the extent of this transmission is so far poorly understood.

In Germany, the national monitoring for zoonotic agents aims at characterizing the prevalence of potential zoonotic pathogens at different stages of various food chains. The monitoring is part of the official control of foodstuffs and fulfills the requirements of EU Directive 2003/99/EC [34]. In 2010, the turkey meat production chain was addressed in this monitoring scheme.

The objective of the present study was to use data from the national monitoring of zoonotic agents in the food chain to obtain a comprehensive insight into the presence and transmission of MRSA in the German turkey meat production chain. A new approach is proposed for analyzing a cross sectional MRSA data set from different stages of the food chain in order to draw conclusions on potential farm to fork transmission. For this purpose, the prevalence of MRSA and the distribution of *spa* types, SCC*mec* types and antimicrobial resistance profiles among MRSA isolated from different steps of the turkey meat production chain were compared. It is proposed that the degree of similarity in the distribution of *spa* types, SCC*mec* types and antimicrobial resistance profiles between the samples from the three process steps may be interpreted as reflecting MRSA transmission along the chain.

## Materials and Methods

### 1. Study Design

Sampling was conducted in 2010 by the competent authorities of the federal states according to a pre-defined protocol in the framework of the national monitoring for zoonotic agents. All participating competent authorities are listed in table S1. Dust samples from 112 German turkey flocks were collected in order to quantify the presence of MRSA in primary production and to assess the introduction of MRSA into the slaughterhouses. Samples at slaughterhouses (n = 359) were analyzed to estimate the transfer to carcasses during slaughter and to determine the transmission of MRSA from carcasses to fresh turkey meat during further processing. Finally, 460 turkey meat portions were sampled to evaluate the MRSA exposure of consumers via contaminated turkey meat.

Turkey pens were sampled by pooling 5 dust swab samples, collected from different sections representing an area of 500 cm^2^, each. At the slaughterhouse, at least 30 g neck skin was sampled from turkey carcasses after slaughter and chilling, but prior to further processing. Samples of 25 g of fresh turkey meat (with or without skin) were collected at retail. In order to ensure a high level of representativity, the distribution of the samples in primary production and at slaughter across Germany was proportional to the number of turkey flocks and the slaughter capacity of the respective federal state. Meat samples at retail were distributed according to the human population size of the executive federal state. A more detailed description of the principles of the national monitoring for zoonotic agents has been published before [35].

### 2. MRSA Isolation

MRSA were isolated by the regional laboratories according to the recommended method of the National Reference Laboratory (NRL) for staphylococci including S. aureus at the Federal Institute for Risk Assessment (BfR). The dust samples were pooled per turkey house in 100 ml Mueller Hinton broth supplemented with 6.5% NaCl for pre-enrichment. Neck skin samples (at least 30 g), fresh meat (25 g) and meat preparations (25 g) were pre-enriched in 225 ml Mueller Hinton broth supplemented with 6.5% NaCl. After incubation for 16–20 h at 37°C, 1 ml pre-enrichment broth was transferred into 9 ml of tryptic soy broth supplemented with 50 mg/l aztreonam and 3.5 mg/l cefoxitin. After incubation of this selective-enrichment broth for a further 16–20 h at 37°C one loopful was plated onto sheep blood agar and chromogenic MRSA screening agar respectively, and incubated for 24–48 h at 37°C. Presumptive MRSA isolates were sent to the National Reference Laboratory (NRL) for staphylococci including *S. aureus* at the Federal Institute for Risk Assessment (BfR) for MRSA confirmation and characterization. The number of MRSA isolates included in further analyses is not exactly congruent to the amount of positive samples obtained within the national monitoring for zoonotic agents because first, the NRL did not always receive the corresponding isolate from the competent authorities of the federal states or second, isolates which did not exactly correspond to the monitoring sampling plan in terms of completeness of data reporting to the national level but were obtained from the correct matrix were excluded from prevalence estimations but included in further typing and strain comparisons.

Twenty one MRSA isolates from wild boar meat within the national monitoring for zoonotic agents of 2011 were used in the analyses as a control group (data not shown in detail). The control group was selected to ensure wide differences with the population under study concerning the distribution of MRSA strains in order to evaluate if the used analytical approach is appropriate to differentiate between the matrices.

### 3. Molecular Typing

Presumptive MRSA isolates were confirmed by an in-house multiplex PCR simultaneously targeting the 23S rDNA specific for *Staphylococcus* species [36], the nuclease gene *nuc* which is specific for *S. aureus*, and the resistance gene *mecA* [37]. Template DNA was extracted using the “RTP Bacteria DNA Mini Kit” (Invitek, Berlin, Germany). All MRSA isolates were further characterized using *spa* typing [38] and SCC*mec*-typing [39]. The method applied for typing of the *SCC*mec differentiates SCC*mec* types I to V and their subtypes. However, isolates of the CC398 characterized as type III by the method have been shown to rather be a variant of type V [40]. The software Ridom Staphytype (Ridom GmbH, Würzburg, Germany) was used to assign *spa* types. *Spa* types which have not been identified and assigned to a clonal complex (CC) by the NRL before were additionally subjected to multilocus sequence typing (MLST) [41].

### 4. Antimicrobial Susceptibility Testing

All isolates were tested for the susceptibility to antimicrobials using broth microdilution in accordance with Clinical and Laboratory Standards Institute guidelines [42]. Commercial microtitre plates were used (TREK Diagnostic Systems, Magellan Biosciences, West Sussex, England). Minimum inhibitory concentrations (MIC) were evaluated according to epidemiological cut-off values (ECOFFs) published for MRSA and *S. aureus* by the European committee for antimicrobial susceptibility testing (www.eucast.org). MIC values above the ECOFFs indicated microbiological resistance. MIC lower or equal to the ECOFFs characterised susceptible strains. *S. aureus* strain ATCC 25923 was used for quality assurance Resistance testing included gentamicin, kanamycin, streptomycin, chloramphenicol, ciprofloxacin, tetracycline, clindamycin, erythromycin, mupirocin, linezolid, vancomycin, quinupristin/dalfopristin, penicillin, fusidic acid, cefoxitin, trimethoprim, sulfamethoxazole, rifampicin and tiamulin.

### 5. Statistical Analysis

The chi square test of homogeneity was used to analyze differences in the distribution of *spa* types and antibiotic resistance profiles between MRSA strains from the turkey flocks, carcasses at slaughter and meat. Isolates were grouped according to their *spa* types and antibiotic resistance profiles to assure appropriate numbers of isolates in all categories. All *spa* types were aggregated in accordance to their frequency of occurrence. The phenotypic antimicrobial resistance profiles were grouped by hierarchical cluster analysis using Ward’s minimum variance and squared Euclidean distance. The MIC values for each isolate were categorized into resistant or susceptible according to the ECOFFs to generate a binary data set. The final amount of clusters was determined using the Pseudo-F [43] and Pseudo-T [44] statistics. Both tests indicate possible breakpoints for splitting the data into the appropriate amount of clusters. The distribution of SCC*mec* types in the different matrices were compared using Fisher’s exact test as 33.3% of the cells of the contingency table had an expected value below 5. P-values of <0.05 were considered statistically significant. Chi square test, Fisher’s exact test and cluster analysis were calculated using the statistical software package SPSS 18.0 (SPSS Inc. Munich, Germany). Pseudo-F and Pseudo-T statistics were performed using SAS/STAT software 9.2 (SAS Institute Inc., Cary, NC, USA).

The degree of similarity between the frequency distributions of *spa* types, SCC*mec* types and resistance profiles of MRSA among the sample sets from the turkey primary production, carcasses at slaughterhouse and turkey meat at retail was estimated using the Czekanowski index or proportional similarity index (PSI) [45]. It is calculated by:
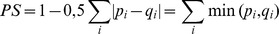
where p_i_ and q_i_ represent the proportion of strains out of all strains among the data sets P and Q which agree in the realization i of the variable of interest. The values for PS range from 1 for identical frequency distributions of the variable of interest to zero for no similarities between the data sets. Since the size of the samples is rather small, a realization of the PSI index may deviate largely from its true value. Thus, the PSI was bootstrapped obtaining a probability density distribution from which we derived the 95% confidence interval for the PSI. The statistic open source software R (available at: http://www.R-project.org) was used to calculate the approximate confidence interval of the Czekanowski index using the non-parametric boostrap BC_α_ method utilizing 2000 iterations [46].

## Results

Twenty two (19.6%) of 112 dust samples from the turkey primary production, 235 (65.5%) of 359 turkey carcasses after slaughter and 147 (32.0%) of 460 turkey meat samples at retail were tested positive for MRSA [47]. A set of 32 isolates from dust samples, 248 isolates from turkey carcasses and 241 isolates from turkey meat was used for further laboratory analyses (Table 1).

**Table 1:** MRSA prevalence and distribution of *spa* types, SCC*mec* types and antimicrobial resistance clusters at different steps of the German turkey meat production chain in 2010.

Process step		Primary production		Slaughter		Meat		total
Samples (n)		112		359		460		931
MRSA positive samples (n)		22		235		147		404
MRSA prevalence (%)		19,6		65,5		32		
No. of isolates included in further statistics^a^		32		248		241		521
Genetic Typing								
CC398	*spa* types	n	%	n	%	n	%	n
	t011	14	43.8	113	45.6	113	46.9	240
	t034	14	43.8	105	42.3	77	32.0	196
	t108	-		-		1	0.4	1
	t571	1	3.1	-		1	0.4	2
	t899	-		1	0.4	5	2.1	6
	t1255	-		-		3	1.2	3
	t1344	-		-		3	1.2	3
	t1580	-		-		1	0.4	1
	t2510	-		-		1	0.4	1
	t2576	-		1	0.4	-		1
	t2970	-		3	1.2	1	0.4	4
	t4652	-		1	0.4	1	0.4	2
	total	29	90.6	224	90.3	207	85.9	460
non CC398								
assigned MLST types	*spa* types							
ST5	t002	1	3.1	14	5.6	22	9.1	37
ST5	t010	-		-		1	0.4	1
ST45	t015	-		-		1	0.4	1
ST9	t1430	2	6.3	10	4.0	10	4.1	22
	total	3	9.4	24	9.7	34	14.1	61
total		32	100	248	100	241	100	521
	SCC*mec*Types							
	n.t.^b^	2	6.3	24	9.7	33	13.7	59
	*mec* III	-		1	0.4	3	1.2	4
	*mec* IVa	7	21.9	47	19.0	65	27.0	119
	*mec* V	23	71.9	176	71.0	140	58.1	339
total		32	100	248	100	241	100	521
	Resistance profiles^c^							
	Cluster A	17	53.1	121	48.8	97	40.2	235
	Cluster B	10	31.3	82	33.1	88	36.5	180
	Cluster C	5	15.6	45	18.1	56	23.2	106
total		32	100	248	100	241	100	521

a MRSA isolates which did not exactly correspond to the monitoring sampling plan in terms of completeness of data reporting to the national level were excluded from prevalence estimations but included in further typing and strain comparisons.

b Not typable.

c Resistance cluster were calculated using Ward’s minimum variance with squared Euclidean distance.

A total of 16 different *spa* types were identified. The number of different *spa* types increased during processing from 5 different types in dust samples over 8 in carcasses to 15 different types in meat samples. The proportion of strains assigned to CC398 ranged between 85.9 and 90.6%. Among CC398, t011 (43.8–46.9%) and t034 (32.0–43.8%) were the predominating *spa*-types on every process step. *Spa* types t1430 (4.0–6.3%) and t002 (3.1–9.1%) were dominating within the group of non CC398 strains.

Most of the strains carried SCC*mec*-type V (58.1–71.9%) followed by type IVa (19–27.0%). Type III (0–1.2%) was identified sporadically (Table 1). However, there is evidence in former literature that CC398 strains which were identified as SCC*mec* type III by the typing scheme of Zhang et al. [39] are rather assigned to a separate variant of SCC*mec* type V [40], [48], [49]. In 5.7–17.6% of the strains the SCC*mec* type could not be identified by the method used.

**Figure 1:**
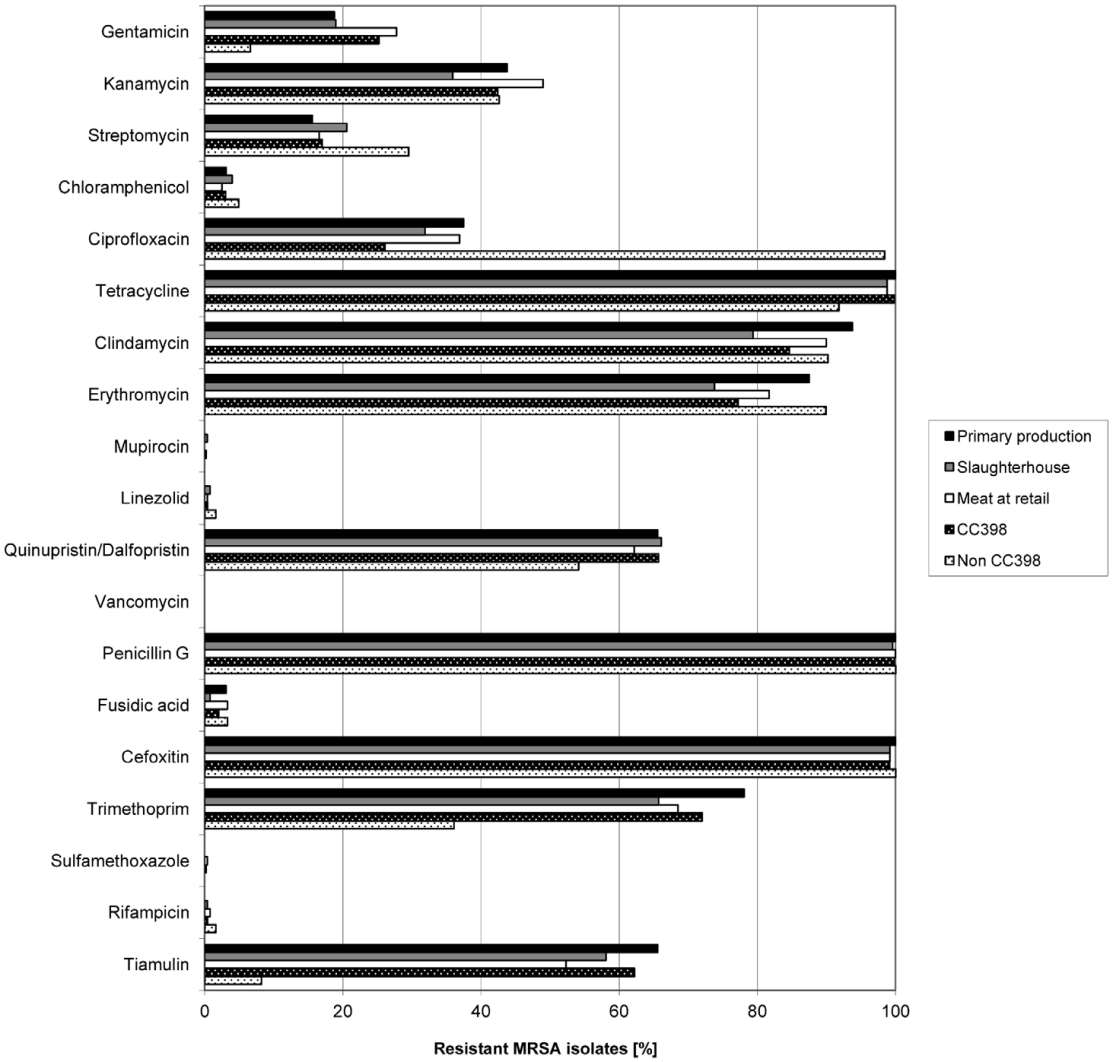
Antimicrobial resistance of MRSA in the German turkey meat production chain. Distribution of antimicrobial resistance of MRSA strains separated into CC398 and non CC 398 strains as well as different steps of the turkey meat production chain isolated from dust samples at turkey primary production (n = 32), carcasses at slaughter (n = 248) and meat at retail (n = 241). The MRSA strains were isolated in the course of the national monitoring for zoonotic agents in Germany in 2010.

Susceptibility to 19 different antimicrobial agents was determined (Figure 1). Throughout the turkey production chain, the vast majority of isolates was resistant to tetracycline (98.8%–100%). High resistance rates were obtained to clindamycin (79.4–93.8%), erythromycin (73.8–87.5%), trimethoprim (65.7–78.1%), quinupristin/dalfopristin (62.2–66.1%) and tiamulin (52.3–65.6%). Resistances to mupirocin, linezolid, sulfamethoxazole and rifampicin were observed sporadically in individual isolates from all steps of the process chain. All isolates were susceptible to vancomycin. Resistance to tiamulin (62.2 versus 8.2%), gentamicin (25.2 versus 6.6%) and trimethoprim (72.0 versus 36.1%) was considerably more frequent among CC398 than among non-CC398 strains. Resistance to ciprofloxacin was common among non-CC398 strains (98.4 versus 26.1% in CC398 strains) (Figure 1).

All 521 MRSA strains were included in further similarity estimations. In accordance to the frequency of their occurrence all *spa* types were aggregated in 4 different categories for further statistical analysis. The most prevalent *spa* types t011 and t034 built their own group whereas rare *spa* types of CC398 and all non CC398 strains were summarized in separate groups. The chi square distribution of the *spa* type groups did not significantly differ between primary production, carcasses at slaughter and meat at retail (p = 0.06). Likewise, no significant difference was identified in the distribution of SCC*mec* types between the origins using fisher’s exact test (p = 0.095). A total of 101 different resistance profiles were identified among the MRSA isolates including resistance to 2 to 12 different antimicrobial substances. The hierarchical cluster algorithm of Wards minimum variance combined with squared Euclidean distance separated the antimicrobial resistance profiles into homogenous clusters. Identical resistance phenotypes did not appear in more than one cluster. Based on the Pseudo-F and Pseudo-T statistics the 3 cluster solution containing 33, 44 and 24 different phenotypic resistance profiles, respectively, was identified to best describe the binary data set. Detailed characteristics of the cluster composition, concerning antimicrobial resistance and the distribution of groups of *spa* types and SCC*mec* types, is summarized in table S2. The antimicrobial resistance clusters did not significantly differ in their chi square distribution between the MRSA samples from the three origins (p = 0.295).

The distribution of *spa* types, SCC*mec* types and antimicrobial resistance profiles within the sample collections from the three process steps and the control group were compared pair wise using the Czekanowski index (Table 2). High index values were obtained for the distribution of *spa* types (PSI 0.79–0.86) among MRSA from the turkey meat chain. The comparison of the distribution of antimicrobial resistance profiles resulted in the lowest index values (PSI 0.42–0.56). The distribution of s*pa* types and antimicrobial resistance profiles showed remarkably higher similarity between the different production steps of the turkey meat chain as to samples from the control group (PSI 0.55–0.56 and 0.13–0.19 resp.). High similarity in the distributions of SCC*mec* types was calculated between all process steps of the turkey meat production chain (PSI 0.85–0.91). However, a strong association was also received with SCC*mec* types of the control group (PSI 0.83–0.85).

**Table 2:** Similarity matrix of *spa* types, SCC*mec* types and resistance profiles of MRSA isolated from the German turkey meat production chain in the course of the national monitoring for zoonotic agents in 2010 (95% confidence intervals).

		Primary production	Slaughterhouse	Meat at retail	Control Group Wild boar meat
		av. PSI^a^ (CI 95%)^b^	av. PSI^a^ (CI 95%)^b^	av. PSI^a^ (CI 95%)^b^	av. PSI^a^ (CI 95%)^b^
Primary production	*spa* types	1			
	SCC*mec* types	1			
	resistance profiles	1			
Slaughterhouse	*spa* types	0.86 (0.72, 0.95)	1		
	SCC*mec* types	0.91 (0.79, 0.98)	1		
	resistance profiles	0.43 (0.30, 0.53)	1		
Meat at retail	*spa* types	0.79 (0.64, 0.90)	0.86 (0.79, 0.92)	1	
	SCC*mec* types	0.85 (0.70, 0.96)	0.87 (0.79, 0.95)	1	
	resistance profiles	0.42 (0.33, 0.51)	0.56 (0.49, 0.62)	1	
Control Group	*spa* types	0.55 (0.33, 0.71)	0.56 (0.38, 0.74)	0.56 (0.38, 0.74)	1
Wild boar meat	SCC*mec* types	0.84 (0.62, 0.98)	0.83 (0.64, 0.96)	0.85 (0.70, 0.95)	1
	resistance profiles	0.13 (0.03, 0.27)	0.19 (0.06, 0.34)	0.14 (0.04, 0.23)	1

a PSI: Czekanowski index or proportional similarity index.

b CI 95%: 95% confidence interval.

## Discussion

In the present study, a new approach is proposed for analyzing a cross sectional set of MRSA isolates originating from three consecutive stages of the turkey meat production chain in order to draw conclusions on a potential farm to fork transmission. In the course of the German national monitoring for zoonotic agents in 2010 MRSA was isolated at all stages of the turkey meat production chain with prevalences ranging from 19.6% to 65.5%. To our knowledge, this is the first representative national MRSA prevalence study in the turkey production chain. In a regional prevalence study among fattening turkeys in southern Germany in 2009, a considerably higher prevalence of 90% MRSA positive flocks was observed using the same sampling procedure [12]. The difference might be explained by the regional restriction of sampling and the small sample size in that study. The proportion of positive meat samples is in line with results from the Netherlands [25]. Outside of Europe, low MRSA contamination rates of 3.85% [32] and 1.7% [50] were reported among US turkey meat.

The high MRSA prevalence in turkey carcasses after slaughter in comparison to the flock prevalence is in contrast to the situation in pigs [12], [51] and indicates that the turkey slaughter process may play an important role in the transmission of MRSA. Turkeys are slaughtered highly automated at a speed of line up to 3,600 turkey hens and up to 2,700 turkey toms per hour which leads to a permanent introduction of MRSA into the poultry processing plants [52]. During the process, MRSA on animal surfaces can get transmitted via direct contact or indirect via surface processing machinery, scalding water or the hands of staff. Scalding takes place at a constant water temperature between 50 and 65°C for 60 to 210 sec [52]. Although the surface of the carcasses is exposed to a heat treatment during scalding, the temperature and duration of the process might be insufficient to substantially reduce superficial MRSA counts. The selective growth of *S. aureus* after the elimination of less heat resistant microbial flora in the scalding water has been discussed [53]. As bacterial counts increase in the tanks throughout the slaughter day scalding can contribute to cross contamination [54]. After scalding, the birds go through the plucking machines consisting of revolving drums with rubber beaters or discs with plucking fingers. The birds are flailed and scraped for 30–90 sec while being sprayed with warm or cold water [52]. Plucking equipment is difficult to clean and a persisting microbiological flora can get established [55]. Cross contamination during slaughter and meat processing might lead to an extensive distribution of *spa* types between different animals and slaughter flocks. In addition, the increase in manual handling during processing facilitates the entry of human MRSA strains into the production units. This can explain the increase in the variability of *spa* types along the chain and is in line with the increase in the proportion of non CC398 strains in meat samples compared to dust or carcasses. *Spa* types t002 and t1430 were also present in primary production and therefore probably have been transmitted along the food chain. In contrast, *spa* types t010, t015 were first observed in meat samples.

The majority of MRSA from the German turkey production chain was assigned to the livestock associated CC398 with the predominant *spa* types t011 and t034. This is in line with results from other livestock like veal calves [9], dairy cattle [56], [57] and pigs [6] as well as in food [25]. In the present study, 37 of the 521 MRSA strains (7.1%) were identified as t002. This *spa* type t002 is assigned to CC5. In Germany, CC5 is one of the epidemic MRSA strains among humans [58]. Finding t002 in turkey flocks and in turkey meat is in line with other studies from central Europe [12], [25], [59]. So far, it is not known, whether this strain originates from the “human” strain and is introduced into the food chain on different levels or whether it got established in the turkey population and is transmitted along the chain. Detailed molecular-epidemiological investigations are needed to compare strains both from human and farm to fork origin. In the present study, 4.2% of the MRSA isolates were characterized as *spa* type t1430, a MRSA strain which was also frequently isolated from chicken meat [25] and broilers at slaughter [60] in the Netherlands. However, it was has also been detected in turkey flocks at farm level [12]. The strain is assigned to ST9, a lineage genetically unrelated to ST398. ST9 is the predominating sequence type among MRSA from pigs in Asian countries [14], [15], [61]–[64]. Outside of Europe, MRSA contamination was reported among US turkey meat [32], [50]. In both surveys, all isolates belonged to USA 300 (ST8), the most common community associated MRSA strain in the USA, suggesting human contamination during processing.

The frequent use of antimicrobials at farm is discussed as a risk factor for the wide dissemination of MRSA in livestock production chains [65]. In recent studies antimicrobials were identified to be used in more than 90% of the investigated turkey flocks and animals received on average 33 daily doses of antimicrobials during raising and fattening [66]. With a share of 21% β-lactams were most often used followed by polypeptides (15.2%), macrolides (13.4%), tetracyclines and aminoglycosides (12.4% both). Fluoroquinolones were used in 6.5% of the investigated flocks. The common application of antimicrobials via drinking water bears the risk of under dosing of individual animals and contamination of the barn environment with antimicrobials which also facilitates the selection of resistance [67].

Cluster analysis was used to better describe the multidimensional data set of antibiotic resistance profiles grouping all MRSA strains within 3 different clusters. As the ordinal MIC values generated by two-fold dilutions in substance concentration are difficult to describe by cluster analysis a binary interpretation of the data set was used. Ward’s minimum variance with squared Euclidian distance was proven to be the best method to produce well separated cluster in binary antimicrobial resistance data sets [68], [69]. No resistance phenotype simultaneously appeared in several clusters. The distribution of *spa* types, SCC*mec* types and the three clusters of antimicrobial resistance types did not significantly differ in the MRSA samples from the three origins. The chi square value was approaching significance with respect to the *spa*-types, which was presumably due to the slightly higher proportion of other CC398 and non CC398. However, considering all three features it cannot be rejected on the basis of the included data that the MRSA isolates from different steps of the turkey meat production chain originate from the same population of strains. This result might rather indicate farm to fork transmission of MRSA of the same pool of strains than development of separate MRSA populations at each step of the chain. The calculation of the Czekanowski index for *spa* type and SCC*mec* type data results in consistently high similarity values between the matrices whereas the comparison of antimicrobial resistance phenotypes observed medium index values. Higher values of similarity were obtained between the adjacent process steps primary production/slaughter and slaughter/meat than between samples from primary production and meat. This result was expected as an increase in the variability of the MRSA isolates might be conceivable at each process stage due to external introduction of new strains via human or environmental contamination or due to spontaneous mutations in the strains.

The lower values of similarity between the distribution of *spa* types and antimicrobial resistance profiles of samples from the turkey meat production chain and the control group indicate that that the proposed statistical method is valid to detect existing differences in the distribution of these characteristics of MRSA.

Concerning SCC*mec* types, high index values were also observed in comparison to the control group which might be explained by the insufficient discriminatory power of SCC*mec* typing. In addition, MRSA isolates with not typeable SCC*mec* cassettes were considered as equal that might lead to an overestimation of similarity.

It can be concluded that MRSA is present at every step of the turkey meat production chain in Germany. Using the Czekanowski index it is possible to quantify the similarity of the distribution of *spa* types, SCC*mec* types and antimicrobial resistance phenotypes between MRSA data sets from different stages of turkey meat production chain. Combined with chi square statistics, the high level of similarity suggests MRSA transmission along the chain.

## Supporting Information

Table S1 List of competent authorities of the German federal states which were responsible for collecting the samples.
(PDF)

Table S2 Distribution of resistance against 19 different antimicrobials, grouped *spa* types and SCC*mec* types within the binary phenotypic resistance clusters of 521 MRSA isolates sampled at different steps of the German turkey meat production chain in 2010.
(PDF)
