# Supplementary material for: Comparison of spa Types, SCCmec Types and Antimicrobial Resistance Profiles of MRSA Isolated from Turkeys at Farm, Slaughter and from Retail Meat Indicates Transmission along the Production Chain
Source: PLoS One. 2014 May 1;9(5):e96308. doi: 10.1371/journal.pone.0096308 (PMC4006815; doi:10.1371/journal.pone.0096308)
Supplement: Table S1 — List of competent authorities of the German federal states which were responsible for collecting the samples. (PDF) [file pone.0096308.s001.pdf]

Table S1:

List of competent authorities of the German federal states which were responsible for collecting the samples

Federal States	Agencies
Baden-Württemberg	Chemical and Veterinary Investigatory Office of Stuttgart
	Chemical and Veterinary Investigatory Office of Karlsruhe
	Chemical and Veterinary Investigatory Office of Freiburg
	Chemical and Veterinary Investigatory Office of Sigmaringen
Bavaria	Bavarian State Office for Health and Food Safety
Berlin/Brandenburg	State Laboratory Berlin Brandenburg
Bremen	State Investigatory Office for Chemistry, Hygiene and Veterinary Medicin
Hamburg	State Institute for Food Safety, Health Protection and Environmental Investigations
Hesse	State Laboratory Hesse
Mecklenburg-Vorpommern	State Office for Agriculture, Food Safety and Fisheries
Lower Saxony	State Office for Consumer Protection and Food Safety
North Rhine-Westphalia	State Veterinary Investigatory Office Arnsberg
	Chemical and Veterinary Investigatory Office Münsterland – Emscher – Lippe
	Chemical and Veterinary Investigatory Office East-Westphalia- Lippe
	Chemical and Veterinary Investigatory Office Rhine-Ruhr-Wupper
Rhineland-Palatinate	State Investigatory Office Koblenz
Saarland	State Office for Consumer Protection
Saxony	State Investigatory Institute for Health and Veterinary Service Saxony
Saxony-Anhalt	State Office for Consumer Protection Halle
Schleswig Holstein	State Laboratory Schleswig Holstein
Thuringia	Thuringia State Office for Consumer Protection
