# Supplementary material for: Comparison of spa Types, SCCmec Types and Antimicrobial Resistance Profiles of MRSA Isolated from Turkeys at Farm, Slaughter and from Retail Meat Indicates Transmission along the Production Chain
Source: PLoS One. 2014 May 1;9(5):e96308. doi: 10.1371/journal.pone.0096308 (PMC4006815; doi:10.1371/journal.pone.0096308)
Supplement: Table S2 — Distribution of resistance against 19 different antimicrobials, grouped spa types and SCCmec types within the binary phenotypic resistance clusters of 521 MRSA isolates sampled at different steps of the German turkey meat production chain in 2010. (PDF) [file pone.0096308.s002.pdf]

Table S2

Distribution of resistance against 19 different antimicrobials, grouped *spa* types and SCC*mec* types within the binary phenotypic resistance clusters of 521 MRSA isolates sampled at different steps of the German turkey meat production chain in 2010.

		Cluster	A		B		C		total	
			n	%	n	%	n	%	n	%
No. of isolates			235	45,1	180	34,5	106	20,3	521	100
No. of resistance profiles			33	32,7	44	43,6	24	23,8	101	-
Antimicrobial substances[a]	GEN	sen	222	55,4	176	43,9	3	0,7	401	77,0
		res	13	10,8	4	3,3	103	85,8	120	23,0
	KAN	sen	150	50,0	150	50,0	0	0,0	300	57,6
		res	85	38,5	30	13,6	106	48,0	221	42,4
	CHL	sen	224	44,4	178	35,3	102	20,2	504	96,7
		res	11	64,7	2	11,8	4	23,5	17	3,3
	CIP	sen	152	44,6	84	24,6	105	30,8	341	65,5
		res	83	46,1	96	53,3	1	0,6	180	34,5
	TET	sen	0	0,0	5	83,3	1	16,7	6	1,2
		res	235	45,6	175	34,0	105	20,4	515	98,8
	CLI	sen	0	0,0	46	59,7	31	40,3	77	14,8
		res	235	52,9	134	30,2	75	16,9	444	85,2
	ERY	sen	4	3,5	73	64,6	36	31,9	113	21,7
		res	231	56,6	107	26,2	70	17,2	408	78,3
	MUP	sen	234	45,0	180	34,6	106	20,4	520	99,8
		res	1	100,0	0	0,0	0	0,0	1	0,2
	LZD	sen	233	45,0	179	34,6	106	20,5	518	99,4
		res	2	66,7	1	33,3	0	0,0	3	0,6
	SYN	sen	0	0,0	96	51,6	90	48,4	186	35,7
		res	235	70,1	84	25,1	16	4,8	335	64,3
	VAN	sen	235	45,1	180	34,5	106	20,3	521	100,0
		res	0	0	0	0	0	0	0	0,0
	STR	sen	189	44,5	155	36,5	81	19,1	425	81,6
		res	46	47,9	25	26,0	25	26,0	96	18,4
	PEN	sen	0	0,0	1	100,0	0	0,0	1	0,2
		res	235	45,2	179	34,4	106	20,4	520	99,8
	FOX	sen	2	50,0	1	25,0	1	25,0	4	0,8
		res	233	45,1	179	34,6	105	20,3	517	99,2
	SMX	sen	235	45,2	180	34,6	105	20,2	520	99,8
		res	0	0,0	0	0,0	1	100,0	1	0,2
	RIF	sen	234	45,2	179	34,6	105	20,3	518	99,4
		res	1	33,3	1	33,3	1	33,3	3	0,6
	FUS	sen	234	45,9	175	34,3	101	19,8	510	97,9
		res	1	9,1	5	45,5	5	45,5	11	2,1
	TIA	sen	0	0,0	124	53,9	106	46,1	230	44,1
		res	235	80,8	56	19,2	0	0,0	291	55,9
	TMP	sen	17	10,1	144	85,7	7	4,2	168	32,2
		res	218	61,8	36	10,2	99	28,0	353	67,8
Spa types	t011		31	12,9	105	43,8	104	43,3	240	46,1
	t034		186	94,9	10	5,1	0	0,0	196	37,6
	other CC398		14	58,3	8	33,3	2	8,3	24	4,6
	non CC398		4	6,6	57	93,4	0	0,0	61	11,7
SCC*mec* Types	III		0	0,0	4	100,0	0	0,0	4	0,8
	IVa		9	7,6	21	17,6	89	74,8	119	22,8
	V		220	65,1	108	32,0	10	3,0	338	64,9
	n.t.		6	10,2	47	79,7	6	10,2	59	11,3

[a] Gentamicin (GEN), kanamycin (KAN), chloramphenicol (CHL), ciprofloxacin (CIP), tetracycline (TET), clindamycin (CLI), erythromycin (ERY), mupirocin (MUP), linezolid (LZD), quinupristin/dalfopristin (SYN), vancomycin (VAN), streptomycin (STR), penicillin (PEN), cefoxitin (FOX), sulfamethoxazole (SMX), rifampicin (RIF), fusidic acid (FUS), ,tiamulin (TIA), trimethoprim (TMP)
